# Current News

**Published:** 1897-04

**Authors:** 


### Current News.

### DENTAL SOCIETY OF THE STATE OF NEW YORK.

The Twenty-ninth Annual Meeting of this Society will be held in Albany, May 12 and 13, at which time the following programme will be presented:

President's Annual Address, H. J. Burkhart, D.D.S.

Report of the Correspondent, R. Ottolengui, M.D.S.

Report of the Committee on Practice, A. R. Starr, D.D.S.

"Amalgam Fillings, with a Practical Demonstration," G. V. Black, M.D., D.D.S., Sc.D., Jacksonville, Ill.

"Dental Organizations," James Truman, D.D.S., Philadelphia.

"Irregularities of the Teeth and their Correction," J. N. Farrar, M.D., D.D.S., New York.

"Cataphoresis," H. W. Gillett, D.M.D., Newport, R. I.

Subject to be announced, B. Holly Smith, M.D., D.D.S., Baltimore.

Members of the profession are fraternally invited to attend.

H. J. Burkhart, D.D.S., Batavia,
*President.*

C. S. Butler, D.D.S., Buffalo,
*Secretary.*

---

### HARVARD ODONTOLOGICAL SOCIETY.

At the Nineteenth Annual Meeting of the Harvard Odontological Society, held at Young's Hotel, February 27, 1897, the following officers were elected: President, Waldo E. Boardman, D.M.D., 184 Boylston Street, Boston; Recording Secretary, Joseph T. Paul, D.M.D., 157 Newbury Street, Boston; Corresponding Secretary, Edward B. Hitchcock, M.D., D.M.D., Newton, Mass.; Treasurer, Lyman F. Bigelow, D.M.D., 194 Marlborough Street, Boston; Editor, Henry L. Upham, D.M.D., 128 Charles Street, Boston.

*Executive Committee.*—Joseph T. Paul, D.M.D., chairman; Frank T. Taylor, D.M.D., William P. Cooke, D.M.D.

Edward B. Hitchcock,
*Corresponding Secretary.*

---

### RECENT PATENTS.

The following is a list of dental patents granted during the past week and reported for the International Dental Journal:

No. 576,593. Dental appliance. H. S. Lowrey, Kansas City, Mo. Filed March 28, 1896.

No. 576,648. Dental surgeon's knife. Wm. Autenrieth, Cincinnati, Ohio. Filed April 27, 1896.

No. 576,654. Dental syringe. W. C. Bunce, Oberlin, Ohio. Filed April 8, 1896.

No. 576,722. Tool-moistening device for dental hand-piece. Wm. Caille, New York City. Filed October 15, 1896.

No. 576,818. Dental spraying apparatus. W. J. McGraw, Walla Walla, Wash. Filed September 23, 1895.

No. 577,063. Dental hand-piece. J. T. Pedersen, Woodside, N. Y. Filed January 6, 1896.

No. 577,064. Slip joint for dental hand-piece. J. T. Pedersen, Woodside, N. Y. Filed March 2, 1896.

No. 577,254. Means for raising and lowering dental chairs. H. E. Hawksworth, Philadelphia, Pa., assignor to S. S. White Dental Manufacturing Co., same place. Filed October 17, 1896.

No. 577,309. Vulcanizer. F. W. Morgan, P. L. Clark, and J. E. Parker, Chicago, Ill., assignor to Rufus Wright, same place. Filed January 31, 1896.

Trade-Mark No. 29,565. Dental antiseptic tablets. Arthur C. Liepe, Milwaukee, Wis.

